# Integrative Analysis of Machine Learning and Molecule Docking Simulations for Ischemic Stroke Diagnosis and Therapy

**DOI:** 10.3390/molecules28237704

**Published:** 2023-11-22

**Authors:** Jingwei Song, Syed Aqib Ali Zaidi, Liangge He, Shuai Zhang, Guangqian Zhou

**Affiliations:** 1 Department of Medical Cell Biology and Genetics, Guangdong Key Laboratory of Genomic Stability and Disease Prevention, Shenzhen Key Laboratory of Anti-Aging and Regenerative Medicine, and Shenzhen Engineering Laboratory of Regenerative Technologies for Orthopaedic Diseases, Health Sciences Center, Shenzhen University, Shenzhen 518060, China; 2050242004@email.szu.edu.cn (J.S.); aqib.ali@email.szu.edu.cn (S.A.A.Z.); liangge_he@163.com (L.H.); 2 Brain Research Centre, Department of Biology, School of Life Sciences, Southern University of Science and Technology, Shenzhen 518055, China; 3 Lungene Biotech Ltd., Shenzhen 518060, China; 4 Senotherapeutics Ltd., Hangzhou 311100, China

**Keywords:** ischemic stroke, machine learning, molecule docking simulations, single-cell transcriptome analysis, nomogram

## Abstract

Due to the narrow therapeutic window and high mortality of ischemic stroke, it is of great significance to investigate its diagnosis and therapy. We employed weighted gene coexpression network analysis (WGCNA) to ascertain gene modules related to stroke and used the maSigPro R package to seek the time-dependent genes in the progression of stroke. Three machine learning algorithms were further employed to identify the feature genes of stroke. A nomogram model was built and applied to evaluate the stroke patients. We analyzed single-cell RNA sequencing (scRNA-seq) data to discern microglia subclusters in ischemic stroke. The RNA velocity, pseudo time, and gene set enrichment analysis (GSEA) were performed to investigate the relationship of microglia subclusters. Connectivity map (CMap) analysis and molecule docking were used to screen a therapeutic agent for stroke. A nomogram model based on the feature genes showed a clinical net benefit and enabled an accurate evaluation of stroke patients. The RNA velocity and pseudo time analysis showed that microglia subcluster 0 would develop toward subcluster 2 within 24 h from stroke onset. The GSEA showed that the function of microglia subcluster 0 was opposite to that of subcluster 2. AZ_628, which screened from CMap analysis, was found to have lower binding energy with Mmp12, Lgals3, Fam20c, Capg, Pkm2, Sdc4, and Itga5 in microglia subcluster 2 and maybe a therapeutic agent for the poor development of microglia subcluster 2 after stroke. Our study presents a nomogram model for stroke diagnosis and provides a potential molecule agent for stroke therapy.

## 1. Introduction

It has been reported that stroke continues to be the second cause of mortality worldwide [1]. Long-term impaired neurological functions after stroke are persistent and profound because of physical disability and high healthcare costs. Stroke diagnosis within the hyperacute phase is challenging and has a high reliance on neuroimaging techniques, resulting in a complicated prehospital diagnosis [2]. Current pharmacological treatment of stroke is limited to only one US Food and Drug Administration approved drug, rt-PA [3].

Microglia, a major components of the cerebral immune system, are the initial responders to ischemic stroke and contribute significantly to early-stage neuroinflammation [4,5]. In the physiological state, resting microglia maintain homeostasis through the phagocytosis of adverse substances and hazardous debris [6]. P2ry12 and Tmem119 are the specific markers for detecting the resting microglia in the brain [7,8,9,10]. During the initial stages of stroke, activated microglia fill the injured area of the ischemic brain [11]. It is reported that highly-activated microglia have several specific markers, including Spp1, Lpl, Lgals3, and Cst7 [12]. Spp1 is enriched in activated microglia and commonly expressed in microglia within the diseased or neurodegenerative CNS [13]. The study of Keren-Shaul defines some signatures of the disease-associated microglia, including Spp1, Lgals3, Lpl, and Cstb [14,15]. Another study also reports that stroke-associated microglia will up-regulate Spp1, Lgals3, and Cstb [16]. Activated microglia exacerbate tissue damage and neuronal death through the overproduction of inflammatory cytokines and chemokines, such as interleukin-1β, tumor necrosis factor-α, nitric oxide, and reactive oxygen species [17]. Depletion of active microglia can diminish infarct volumes and enhance neurological outcomes [18]. Inhibiting the secretion of inflammatory factors by microglia can alleviate neuronal impairment [19]. These findings indicate that manipulating the activation of microglia and following inflammation is a therapeutic strategy for brain repair.

Bulk RNA-seq is widely used to measure the average expression of genes and capture genome-wide transcriptomic profiles from diverse tissues. However, it hides cell type-specific signature genes and conceals cellular subclusters with different disease response levels [20]. In contrast, single-cell RNA sequencing (scRNA-seq) technology allows investigators to identify the cell subclusters during dynamic biological transcription at single cell resolution. RNA velocity and pseudo time analysis, which are advanced algorithms utilized in scRNA-seq, can facilitate the mapping of cell trajectories throughout dynamic processes, such as clonal evolution, cell differentiation, and cell state transition [21]. The scRNA-seq technique offers an occasion to infer the driver genes and provide deeper insights into the transcriptional alterations associated with pathological development [22,23]. It can also provide new insights into therapeutic strategies such as drug target finding at single cell level [24].

Molecular docking involves the potential interactions between two or more molecules, typically a protein and a small molecule. In the context of drug discovery, molecular docking has emerged as a pivotal tool, especially for the virtual screening of prospective therapeutic agents [25,26]. In recent years, the domain of molecular diagnostics has witnessed remarkable advancements due to the application of machine learning techniques. These techniques provide potent instruments for analyzing complex biological data and extracting valuable insights. Among the various machine learning algorithms, LASSO (Least Absolute Shrinkage and Selection Operator), SVM-RFE (Support Vector Machine Recursive Feature Elimination), and Boruta have garnered considerable acclaim for their effectiveness in feature selection and classification tasks in molecular diagnostics [27,28,29]. However, the majority of extant studies predominantly concentrate on the singular exploration of machine learning or molecular docking, with a notable dearth of attention devoted to the concurrent application of these methodologies in the context of ischemic stroke diagnosis and therapy.

In the present study, we employed weighted gene coexpression network analysis (WGCNA) to ascertain gene modules associated with stroke and used the maSigPro R package to seek the time-dependent genes in the progression of stroke. Three machine learning algorithms (LASSO, SVM-RFE, and Boruta) were further employed to identify the feature genes of stroke. A nomogram model was built based on these feature genes. Analysis of scRNA-seq data was performed to discern microglia subclusters. The RNA velocity and pseudo time analysis were used to infer the transcriptional and functional changes among microglia subclusters after stroke. Connectivity map (CMap) analysis and molecule docking were applied to screen a potential therapeutic agent for stroke. Our work aimed to investigate a nomogram model for stroke diagnosis and provide a potential molecule agent for stroke therapy.

## 2. Results and Discussion

### 2.1. Biomarkers and Nomogram for Ischemic Stroke Diagnosis

It has been reported that stroke continues to rank as the second leading cause of mortality on a global scale [1]. Long-term impaired neurological functions after stroke are persistent and profound because of physical disability and high healthcare costs [30]. Due to the narrow therapeutic time window of acute ischemic stroke, merely a limited fraction of patients can benefit from endovascular treatment [31]. It was demonstrated that the outcome of stroke highly depended on the time from stroke onset to treatment [32]. Stroke patients will get better outcomes and greater reduction of stroke risks with earlier clinical detection and treatment. Thus, there is an imperative necessity to ascertain potential biomarkers and build a model to improve the ischemic stroke diagnosis. Therefore, we employed WGCNA to identify the gene modules associated with stroke and the maSigPro R package to seek the time-dependent genes in the peripheral blood after stroke. Three machine learning algorithms (LASSO, SVM-RFE, and Boruta) were further used to ascertain the feature genes of stroke.

#### 2.1.1. Weighted Correlation Network Analysis and Time-Dependent Genes in the Peripheral Blood of Ischemic Stroke

**Figure 1:**
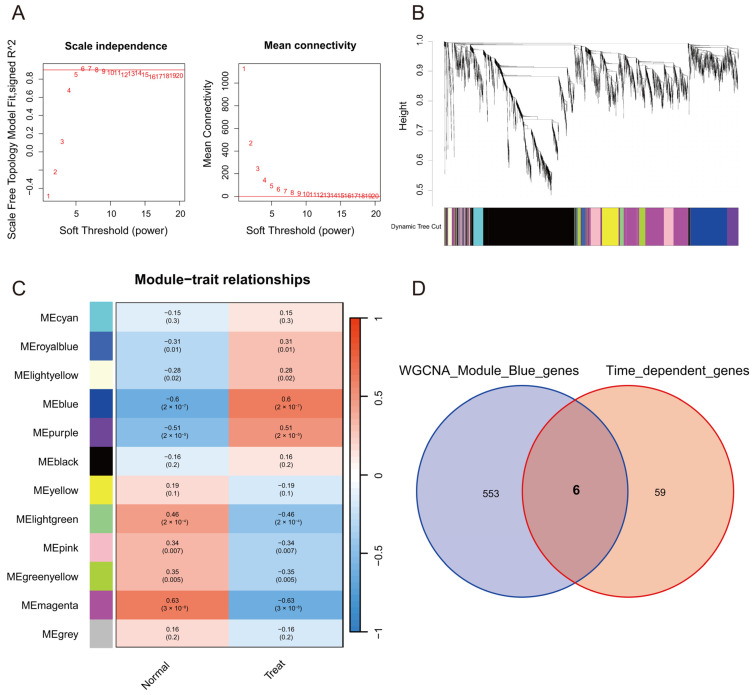
Weighted correlation network analysis (WGCNA) in GSE16561. (**A**) Relationship between the scale-free fit index and a range of soft-thresholding powers (**left**). Association between mean connectivity and diverse soft-thresholding powers (**right**). The scale-free fit index measures the degree of scale-free topology in the network. The mean connectivity indicates the overall connectivity pattern and density of the network. The soft-thresholding power is used to transform the co-expression similarity matrix into a weighted adjacency matrix. The results show that 6 is the optimal soft-thresholding power for the scale-free topology in the network. (**B**) Clustering dendrogram showing co-expression modules identified by WGCNA. (**C**) Heatmap showing the module-trait correlations and corresponding *p* values. (**D**) Venn diagram showing the six overlapping genes between WGCNA screened genes and maSigPro selected genes.

WGCNA can combine genes with clinical trait data and construct a gene co-expression network to identify several highly correlated signature modules that may be involved in critical biological processes. To further analyze the highly correlated genes of stroke, we employed the WGCNA method to identify the critical modules by analyzing the expression profile data from the GSE16561 dataset, which contained 39 stroke patients and 24 normal subjects. We calculated the network topology using soft-thresholding powers from 1 to 20 and set the optimal threshold at 6 (Figure 1A). Furthermore, 12 modules were identified by the hierarchical clustering dendrogram (Figure 1B,C). Among these modules, the blue module had good correlations with stroke (*p* < 0.001). Finally, 559 genes were obtained in the blue module. Time-dependent genes were identified using the maSigPro R package. The results indicated that 65 genes displayed significant temporal fluctuations in the peripheral blood. To acquire marker genes that depict temporal fluctuations within 24 h from stroke onset, we intersected the 559 genes screened by WGCNA and the 65 genes selected by maSigPro. Six overlapping genes were obtained, including Ankrd13a, Cop1, Entpd1, Gyg1, Tcirg1, and Sh3glb1 (Figure 1D).

#### 2.1.2. Identification of Diagnostic Biomarkers and Construction of Clinical Nomogram for Ischemic Stroke

**Figure 2:**
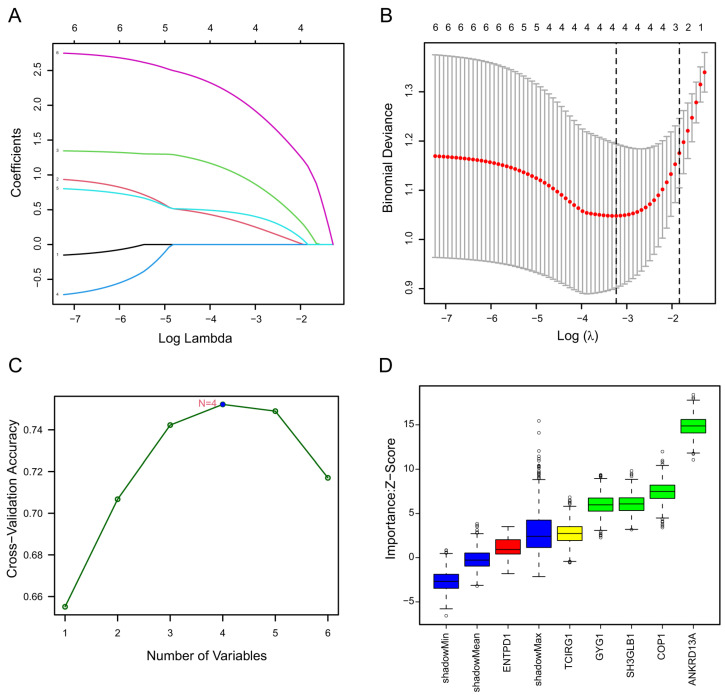
Identification of stroke biomarkers by machine learning. (**A**) Least Absolute Shrinkage and Selection Operator (LASSO) coefficient profiles at varying levels of penalization. (**B**) Screening of the optimal lambda with fivefold cross-validations. (**C**) A line graph depicting the sequential stages involved in biomarker screening by the Support Vector Machine Recursive Feature Elimination (SVM-RFE) algorithm. (**D**) Four genes with importance ranking were determined via the Boruta algorithm.

**Figure 3:**
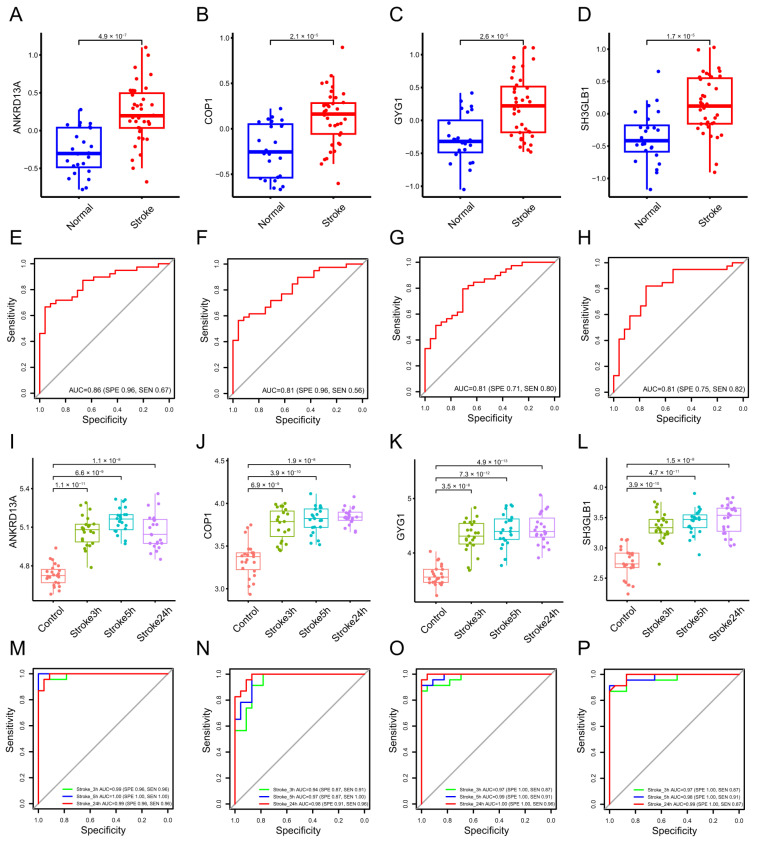
Classification performance of Ankrd13a, Cop1, Gyg1, and Sh3glb1 in GSE16561. (**A**–**D**) The box plots present the expression levels of the four feature genes between normal subjects and stroke patients in GSE16561. (**E**–**H**) Diagnostic efficiency evaluation of the four feature genes by receiver operating characteristic (ROC) curve in GSE16561. (**I**–**L**) The box plots show the expression levels of the four feature genes between normal subjects and stroke patients in GSE58294. (**M**–**P**) Diagnostic efficiency evaluation of the four feature genes by ROC curve in GSE58294.

LASSO regression can be performed on the selected genes to solve the multicollinearity problems, thus reducing the number of candidate genes [33]. The SVM-RFE algorithm is one of the most supervised machine learning methods to handle high dimensional data [34]. The Boruta algorithm is a supervised classification method utilized for feature selection, aiming to ascertain features associated with a given classification task [35]. These methods are widely used to identify biomarkers with superior accuracy and good interpretability [27,28,29]. They were employed to obtain feature genes of stroke. LASSO regression analysis selected four signature genes, including Ankrd13a, Cop1, Gyg1, and Sh3glb1. SVM-REF and Boruta algorithms also indicated that these feature genes have the best classification performance (Figure 2A–D). Box plots were utilized to show the comparison of the expression levels of the four screened signatures between normal subjects and stroke patients. The results showed that these screened signatures were significantly upregulated in the peripheral blood of stroke patients in the GSE16561 and GSE58294 datasets (*p* < 0.0001, Figure 3A–D,I,J). To evaluate the predictive power of these feature genes in the diagnosis of patients with stroke, we performed ROC analyses in both the training and validation cohorts. The results showed that the AUC values of Ankrd13a, Cop1, Gyg1 and Sh3glb1 were 0.86 (95% CI, 0.70–0.92), 0.81 (95% CI, 0.70–0.91), 0.81 (95% CI, 0.70–0.91) and 0.81 (95% CI, 0.77–0.95) in the training set GSE16561, respectively (Figure 3E–H). The results of the validation set GSE58294 also showed that Ankrd13a, Cop1, Gyg1, and Sh3glb1 had better performances with all of the AUC values larger than 0.90 (Figure 3M–P). The above results suggested that these feature genes were robust to the discrimination of patients with stroke.

**Figure 4:**
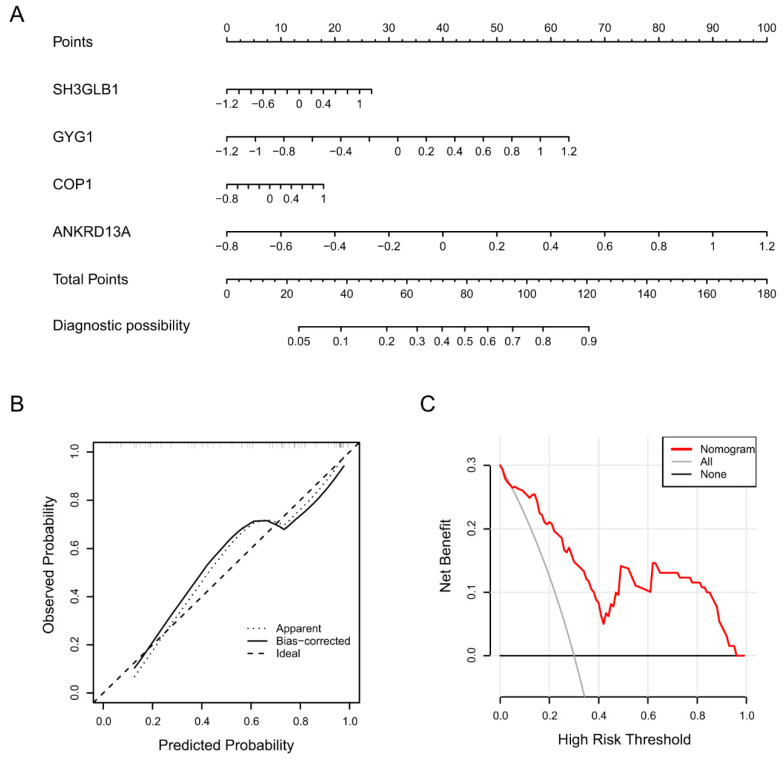
Nomogram model for predicting patients with stroke. (**A**) The visible nomogram was constructed using the four screened feature genes. (**B**) Calibration curve of the nomogram model. The *x*-axis portrays the predicted stroke risk. The *y*-axis illustrates the actual diagnosed stroke. (**C**) decision curve analysis (DCA) curves of the nomogram model. The black line represents the net benefit where none of the participants are assumed to suffer from stroke. Conversely, the light gray line represents the net benefit when all participants are assumed to suffer from stroke. The region bounded by the red line and the light gray line in the model curve signifies the clinical utility of the nomogram model.

Based on the above four feature genes, we established an intuitional nomogram for stroke diagnosis using the rms R package (Figure 4A). The mathematical formula of this model is In(p/(1 − p)) = 0.727 + 0.594 × Sh3glb1 + 1.403 × Gyg1 + 0.529 × Cop1 + 2.659 × Ankrd13a. In this formula, p is the probability of the risk, and In is the natural logarithm. The calibration curve illustrated that the predicted stroke risks were very close to the real stroke risks, indicating that the nomogram facilitated precise assessment of patients with stroke (Figure 4B). Furthermore, DCA indicated that the nomogram model offered a better clinical benefit (Figure 4C). The above results suggested that the established nomogram model has high practical utility and good diagnostic efficacy in the discrimination of stroke.

Ankrd13a, also known as Ankyrin repeat domain-containing protein 13A, is a protein whose specific role in stroke pathophysiology is not well-established. A recent study demonstrated that Ankrd13a is a newly identified regulator within the early stage of the cell-death checkpoint. Elevated Ankrd13a expression exhibits an inverse correlation with apoptotic phenotypes observed in ovarian cancer and is linked to a poor prognosis [36]. Ankrd13a shows distinct alternative splicing patterns in the whole blood transcriptomes of patients with ischemic stroke [37]. Experimental evidence indicates that Ankrd13a upregulates in the brain tissues of ischemic stroke mice compared with those in normal mice [38,39]. Cop1, severed as an E3 ubiquitin ligase, has been acknowledged as a crucial regulator in diverse biological processes, such as cell growth, DNA repair, and apoptosis. Cop1 exerts a suppressive effect on microglia activation and neuroinflammation by facilitating ubiquitin-mediated degradation of C/EBPβ, thereby alleviating I/R-induced injury in stroke [40]. While Gyg1 may not have a well-defined role in stroke, it is reported that Gyg1 is involved in glycogen synthesis in muscle. Expression of mutated Gyg1 in the heart can result in abnormal glycogen accumulation and cardiomyopathy development [41]. According to recent studies, abnormal glycogen accumulation is observed in ischemic stroke patients after reperfusion [42]. Glycogenolytic dysfunction in astrocytes is primarily responsible for this phenomenon. This dysfunction inhibits the breakdown of glycogen into glucose-6-phosphate, consequently leading to a reduction in glucose-6-phosphate concentrations during the ischemic reperfusion (I/R) process. Glucose-6-phosphate is capable of participating in the pentose phosphate pathway to produce glutathione and nicotinamide adenine dinucleotide phosphate (NADPH), both of which are vital antioxidants in cells. Therefore, the decline in glucose-6-phosphate levels results in an escalation in the production of reactive oxygen species (ROS), which exacerbates oxidative damage and hinders the repair process of the brain after ischemic stroke [43]. Sh3glb1, also known as endophilin B1, exerts its proapoptotic function in non-neuronal cells through the facilitation of mitochondrial fragmentation and the activation of Bax and Bak. Nevertheless, Sh3glb1 deficient mice exhibited augmented astrogliosis response and larger infarct volumes following ischemic stroke [44]. Additional investigation is needed to unravel the precise role of the four genes mentioned above in ischemic stroke in future studies.

### 2.2. The Role of Microglia Subclusters in Ischemic Stroke

Microglia undergo metabolic and phenotypic shifts to maintain tissue homeostasis or respond to acute or chronic stress. The role of microglia subclusters in the pathologic progress of stroke remains unclear. Therefore, we aim to elucidate this issue through the application of scRNA-seq analysis.

#### 2.2.1. Identification of Microglia Subclusters and Their Dynamic Changes in Ischemic Stroke

**Figure 5:**
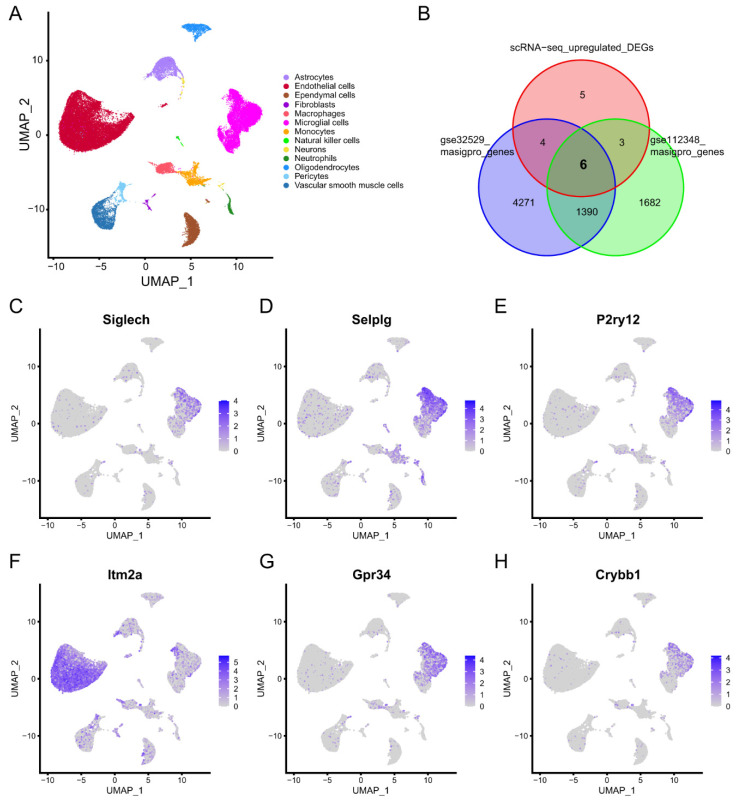
Single-cell RNA sequencing (scRNA-seq) profiles of normal and ischemic stroke mouse brains. (**A**) Uniform manifold approximation and projection (UMAP) plot showing the 13 identified cell types in scRNA-Seq. (**B**) Venn plot showing six overlapping genes between the scRNA-seq upregulated differentially expressed genes (DEGs) and time-dependent genes. (**C**–**H**) Feature plot showing the expression of the six overlapping genes.

A total of 53,218 cells from the GSE174574 database passed quality control and were retained for further analysis. Thirteen cell types were annotated using the SingleR R package and CellMarker Database (Figure 5A). The differentially expressed genes (DEGs) between cells in normal mice and in stroke mice were calculated by the FindMarkers function of Seurat. Time-dependent genes in the cerebral cortex of GSE32529 and GSE112348 were identified using the maSigPro R package. Six overlapping genes were identified between the scRNA-seq upregulated DEGs and the time-dependent genes (Figure 5B). We found that five of the six overlapping genes were highly expressed in microglia cells (Figure 5C–H). These results suggested that microglia play a significant role in the pathological process within 24 h after stroke onset.

**Figure 6:**
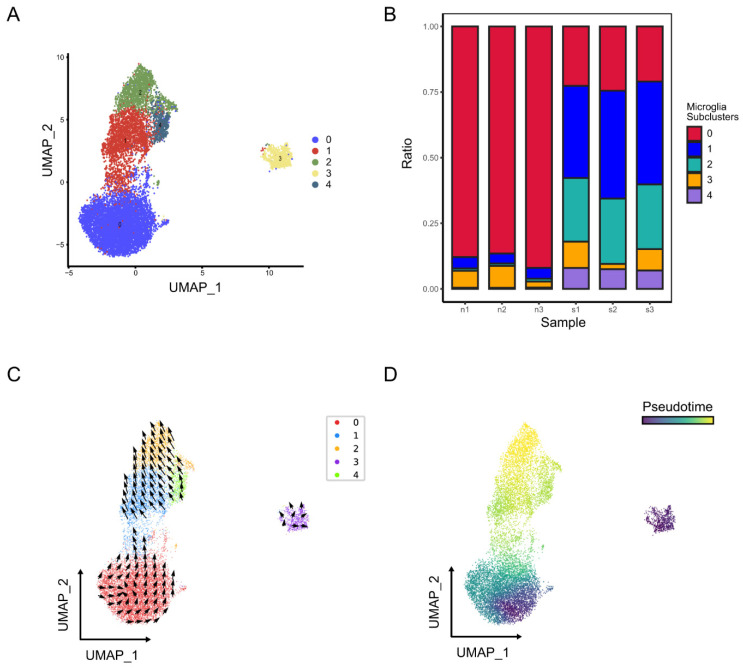
Identification of microglia subclusters in normal and ischemic stroke mouse brains. (**A**) UMAP plot exhibiting the five microglia subclusters. (**B**) The relative proportion of each subcluster of microglia in normal and ischemic stroke mouse brains. The RNA velocity (**C**) and pseudo time (**D**) of microglia are shown in the UMAP plots.

Microglia were further classified into five subclusters (Figure 6A). As shown in Figure 6B, subcluster 2 of microglia was highly concentrated in the brains of stroke mice. The trajectory and pseudo time analysis show that subcluster 0 was in the early stage of the trajectory, followed by subclusters 1 and 4, which were positioned in the intermediate state, and finally reached a terminal state of subcluster 2 (Figure 6C,D). Our results also indicated that microglia subcluster 0 highly expressed the resting microglia markers P2ry12 and Tmem119 [7,8,9,10], while microglia subcluster 2 highly expressed the activated microglia markers Spp1, Lpl, Lgals3 and Cstb [12,13] (Table S1). These results revealed that microglia changed their transcriptional status from a homeostatic state to an activated state after stroke.

#### 2.2.2. GSEA of the Microglia Subclusters 0 and 2

**Figure 7:**
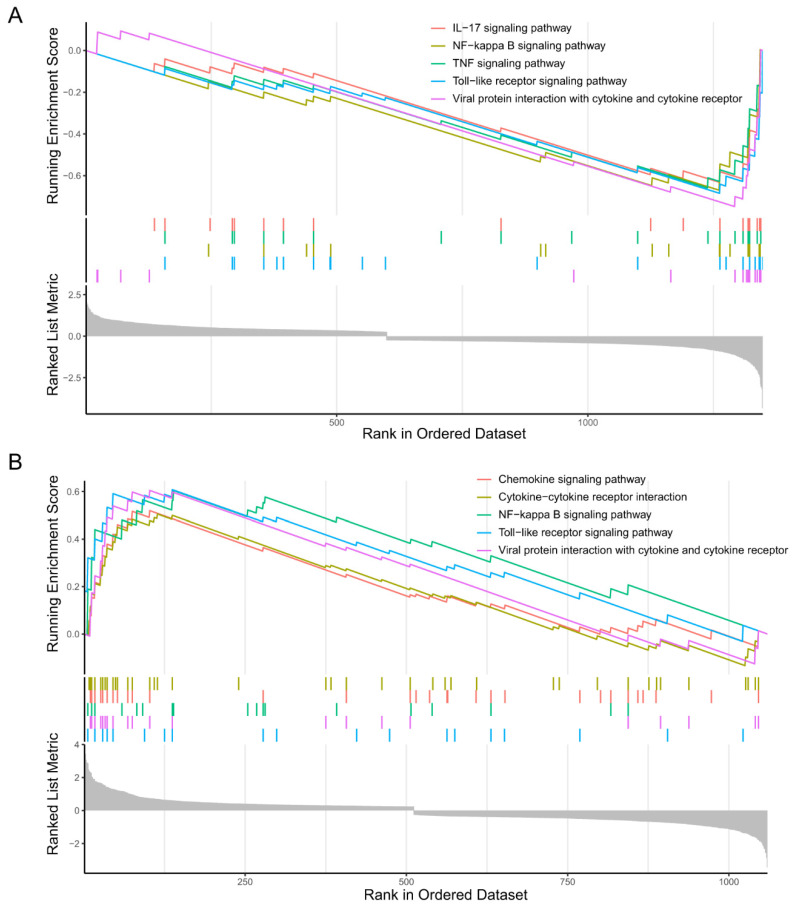
Gene set enrichment analysis (GSEA) of the microglia subclusters 0 and 2. (**A**) GSEA plot showing the top five negatively enriched biological processes of microglia subcluster 0 with the running enrichment score and positions of gene set members on the rank-ordered marker gene list. (**B**) GSEA plot showing the top five positively enriched biological processes of microglia subcluster 2 with the running enrichment score and positions of gene set members on the rank-ordered marker gene list.

The GSEA results indicated that microglia subcluster 0 down-regulated the IL-17 signaling pathway, NF-kappa B signaling pathway, TNF signaling pathway, and Toll-like receptor signaling pathway (Figure 7A). On the contrary, microglia subcluster 2 upregulated the cytokine- cytokine receptor interaction, chemokine signaling pathway, NF-kappa B signaling pathway, and Toll-like receptor signaling pathway (Figure 7B).

It was reported that activated microglia aggravated tissue damage and neuroinflammation by overproduction of inflammatory cytokines and chemokines [45]. NF-kappa B has been recognized as the vital molecule leading to microglia activation, involving inflammatory responses after cerebral ischemia and inducing subsequent neuronal cell death [46,47,48]. Toll-like receptor 2 and Toll-like receptor 4 could activate microglia, resulting in pro-inflammatory cytokine production and brain injury after ischemic stroke [49]. These results showed that the function of microglia subcluster 0 was opposite to that of microglia subcluster 2. According to the previous studies mentioned above, it is reasonable to speculate that microglia subcluster 0 develops toward microglia subcluster 2 within 24 h from stroke onset and will cause subsequent stroke damage.

### 2.3. Molecule Docking Simulations for Ischemic Stroke Therapy

#### 2.3.1. Key Genes of Microglia Subcluster 2

**Figure 8:**
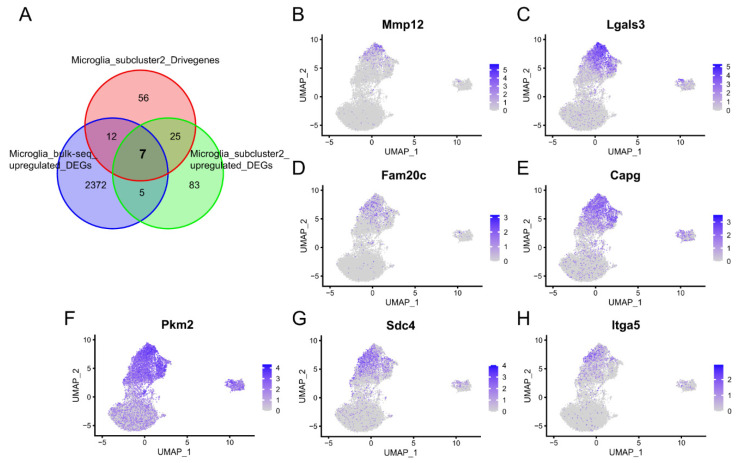
The key genes of microglia subcluster 2 that can not only be detected at the bulk RNA-Seq level but can also be examined at the scRNA-Seq level. (**A**) The Venn diagram illustrates the intersection of seven genes present in both the upregulated DEGs obtained from bulk RNA-Seq analysis and the upregulated DEGs and driver genes identified within microglia subcluster 2. (**B**–**H**) Feature plot showing the expression of the seven overlapping genes.

In order to find the key genes of microglia subcluster 2 that can not only be detected at the bulk RNA-Seq level but can also be examined at the scRNA-Seq level, we collected a dataset PRJNA687414 containing bulk RNA-Seq data of microglia cells. Subsequently, we intersected the upregulated DEGs of bulk RNA-Seq with the upregulated DEGs and driver genes of microglia subcluster 2. Finally, seven overlapping genes were obtained, including Mmp12, Lgals3, Fam20c, Capg, Pkm2, Sdc4, and Itga5 (Figure 8A). The UMAP plot showed that these genes were explicitly highly expressed in microglia subcluster 2 (Figure 8B–H).

#### 2.3.2. Connectivity Map Analysis Identified Potential Candidate Compounds Targeting Microglia Subcluster 2

Because microglia were implicated in the pathological processes of stroke and the transcriptional state of microglia would develop from subcluster 0 to subcluster 2 within 24 h after stroke onset, we tried to screen small potential compounds that target microglia subcluster 2.

**Figure 9:**
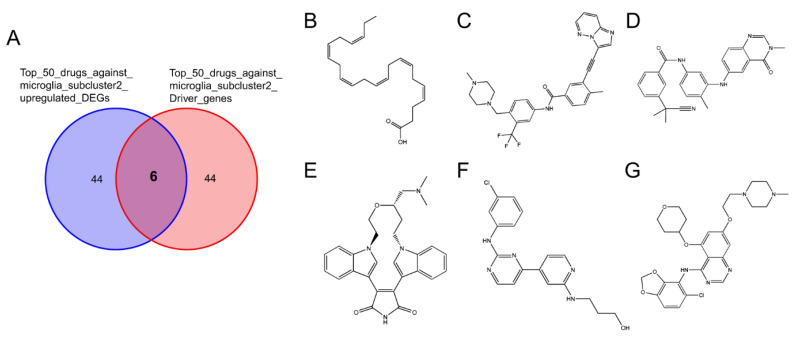
Connectivity map analysis. (**A**) Venn plot showing the six potential candidate compounds targeting microglia subcluster 2. The 2D structures of doconexent (**B**), ponatinib (**C**), AZ_628 (**D**), ruboxistaurin (**E**), CGP-60474 (**F**), and saracatinib (**G**).

CMap was utilized to identify novel potential targets by establishing associations between patterns of gene expression and diseases. The drugs exhibited negative enrichment values, suggesting their potential to reverse dysregulated gene expression and ameliorate the pathological condition [50]. We uploaded 120 upregulated DEGs and the top 100 driver genes of microglia subcluster 2 to the CLUE database (Table S2). Six overlapping compounds were obtained from the intersection between the top 50 compounds against 120 upregulated DEGs and the top 50 compounds against driver genes of microglia subcluster 2 (Figure 9A). The 2D structures of the six overlapping compounds, including doconexent, ponatinib, AZ_628, ruboxistaurin, CGP-60474, and saracatinib, are shown in Figure 9B–G.

#### 2.3.3. Potential Therapeutic Agents Predicted by Molecule Docking

**Figure 10:**
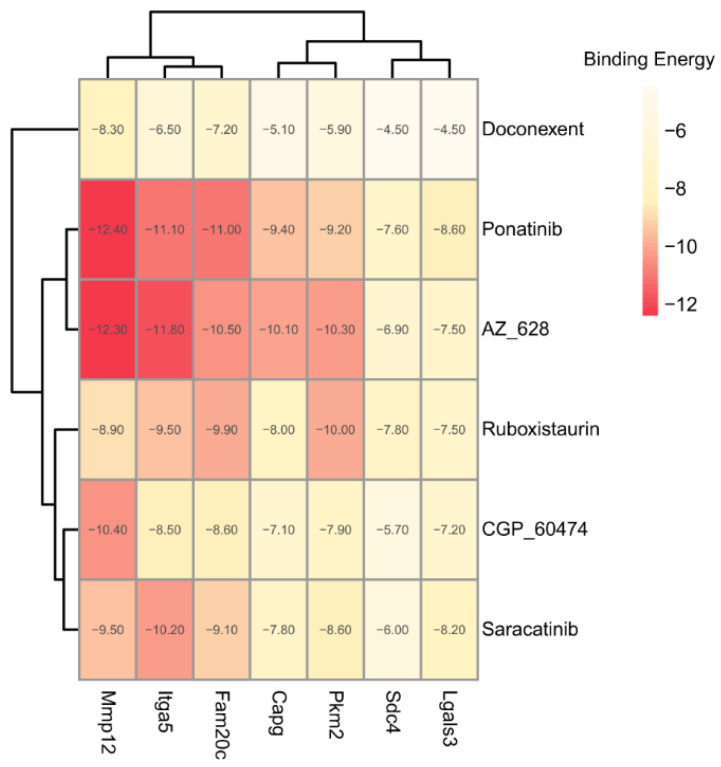
Heatmap showing the binding energy between the six screened compounds and the seven target proteins.

**Figure 11:**
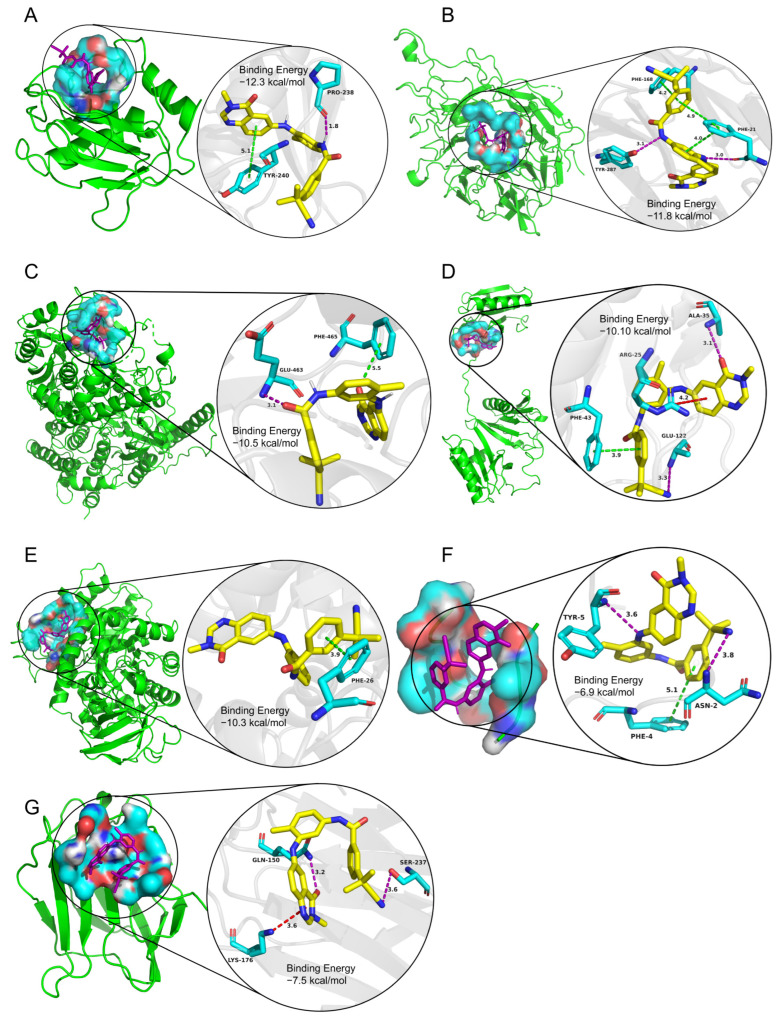
The interactions in the binding pockets between molecule AZ_628 and the seven target proteins were visualized by PyMol software (v2.5.5). (**A**) Mmp12, (**B**) Itga5, (**C**) Fam20c, (**D**) Capg, (**E**) Pkm2, (**F**) Sdc4, and (**G**) Lgals3.

The binding energies between the ligands and receptors were calculated using AutoDock-Vina software (v1.1.2). The lower binding energy corresponds to a more stable binding conformation. A binding energy lower than −5.00 kcal/mol indicates a favorable binding strength. Interestingly, we found that the six screened compounds had low binding energy with the above seven key proteins of microglia subcluster 2 (Figure 10). Especially, molecule AZ_628 had lower binding energy and could form hydrogen bonds, Pi-Pi stacking interactions, or Cation-Pi interactions with them. The interactions in the binding pockets between molecule AZ_628 and the above seven proteins were visualized by PyMol software (v2.5.5) (Figure 11A–G).

AZ-628, a newly identified pan-Raf inhibitor, has exhibited promising efficacy in vascular remodeling and cancers [51,52]. It was reported that AZ-628 could mitigate chondrocyte inflammation and microenvironment deterioration by exerting inhibitory effects on the NF-κB signaling pathway and suppressing RIP3 activation [53]. In addition, our GSEA results also showed that microglia subcluster 2 upregulated the NF-κB signaling pathway. These findings implied that AZ-628 maybe a therapeutic agent for the poor development of microglia subcluster 2 after stroke.

### 2.4. Limitations

There are several limitations in the present study. First, the samples should continue to be collected and additional independent validation cohorts are essential to establish model robustness in future studies. Second, feature selection has always been challenging because the selected features tend to vary significantly according to the method applied. Our findings should be interpreted within the scope and limitations of the methodology used. Lastly, future studies should include cellular or animal experiments to further explore the potential molecular mechanism of the biomarkers, confirm the results of molecular docking, and investigate the therapeutic effects of the predicted drugs.

## 3. Materials and Methods

### 3.1. Data Collection and Settings

All relevant scRNA-seq, microarray data, and RNA-seq data for this retrospective study can be accessed in the GEO database (https://www.ncbi.nlm.nih.gov/geo/, accessed on 1 March 2023) or the EMBL-EBI database (https://www.ebi.ac.uk/, accessed on 1 March 2023). Table 1 shows the details of the collected data.

### 3.2. scRNA-Seq Data Analysis

Count data were obtained from the NCBI GEO database with accession ID GSE174574 and were further processed by the Seurat R package (v4.3.0). Briefly, we filtered out cells with the number of expressed genes lower than 500 or larger than 6000, cells with more than 25% of total genes corresponding to ribosomal genes, and cells with a percentage of mitochondrial genes over 10% of total genes. Simultaneously, genes expressed in less than three cells were omitted. Then, the gene expression matrix was logarithmically normalized and scaled. Principal components analysis (PCA) was conducted with the 2000 genes exhibiting the highest variability. The Harmony R package (v0.1.1) was used to adjust the batch effects. The identified clusters were visualized on a uniform manifold approximation and projection (UMAP) plot. The Seurat FindAllMarkers function was utilized to identify the DEGs. Finally, we annotated the clusters using the singleR R package (v1.0) followed by manual correction with the CellMarker database.

### 3.3. MicroArray Data Processing

The expression profile matrix and the related platform annotation documents were acquired from the GEO database. We mapped the probe sequences to the human genome (GRCh38/hg38) and transformed them into corresponding gene symbols. Probes lacking annotations or containing annotations for multiple genes were excluded from the analysis. In addition, we only reserved the probes with the highest mean value in cases where multiple probes were mapped to the same gene. Finally, the expression profile matrix went through further processing followed by log2 transformation and quantile normalization by the limma R package (v3.54.2).

### 3.4. RNA-Seq Data Processing

Raw sequence data underwent quality control using FastQC, and then the reads were trimmed using Trim_galore. Subsequently, reads were mapped to the mouse genome (GRCm39) using Hisat2. The quantification of gene expression counts was performed using the featureCounts software (v2.0.3). Differential expression genes were calculated using DESeq2 with the adjusted *p* < 0.05 and absolute log_2_(fold-change) > 1.

### 3.5. Weighted Gene Coexpression Network Analysis

The WGCNA R package (v1.72-1) was applied for weighted correlation network analysis. Genes with a high variance of 25% were selected for further analysis. Next, the soft-thresholding powers from 1 to 20 were set in the pickSoftThreshold function of WGCNA to seek the optimal threshold. The optimal thresholding power, herein designated as 6, was subsequently employed to build the adjacency matrix. Then, the topological overlap matrix, which was created from the adjacency matrix, was used to evaluate the connectivity of the network. Imposing a minimum module size of 50, hierarchical clustering was employed to create module dendrograms. The association between each module and clinical traits was assessed by the gene significance and the module membership.

### 3.6. Time-Dependent Genes in the Progression of Stroke

Time-dependent genes were calculated using normalized expression values by the maSigPro R package (v1.70.0). Briefly, the p vector function was employed to calculate a regression fit for each gene. The identification of temporally DEGs was facilitated through the generalized linear model setting with *p* ≤ 0.05 after false discovery rate correction. Then, stepwise regression, with the r-squared parameter set to 0.6, was employed to ascertain the optimal combination of independent variables and screen the statistically significant genes.

### 3.7. Screening of Diagnostic Biomarkers for Stroke

LASSO regression was conducted using the glmnet R package (v4.1-7) with 10-fold cross-validation. The SVM-RFE algorithm was conducted using the e1071 R package (v1.7-13) with a radial basis function kernel and 10 repeats of 5-fold cross-validation. Concurrently, the Boruta algorithm, facilitated by the Boruta R package (v8.0.0), was applied to select the features labeled with “Confirmed” through 10 repeats of 5-fold cross-validation. These three machine learning algorithms were performed on the GSE16561 dataset. The overlapping genes were then obtained by intersecting the LASSO identified genes, SVM-REF screened genes, and Boruta selected genes. To assess the classification performance of the selected biomarkers, we conducted receiver operating characteristic (ROC) analysis in training cohort GSE16561 and calculated AUC values via the reportROC R package (v3.6). The diagnostic biomarkers were also validated in another external cohort GSE58294. When the AUC exceeds the threshold of 0.7, it is deemed to possess diagnostic efficacy.

### 3.8. Nomogram Construction and Evaluation

A nomogram was created for the screened feature genes using the rms R package (v6.7-1). A Calibration curve was generated to evaluate the predictive accuracy. Meanwhile, decision curve analysis (DCA) was utilized to assess the overall utility of the nomogram within a clinical setting for the purpose of evaluating net benefit.

### 3.9. RNA Velocity and Pseudo Time Analysis

The scRNA-seq data was processed by the Cellranger software (v7.1.0). The spliced and unspliced transcript counts were estimated by the scVelo software (v0.2.5). The driver genes of each cell cluster were calculated by the tl.rank_dynamical_genes function of scVelo. Finally, the RNA velocity and pseudo time were evaluated by the CellDancer software (v1.1.7).

### 3.10. Gene Set Enrichment Analysis (GSEA)

DEGs of microglia subclusters were recognized by the Seurat Findallmarker function. To identify the biological process enriched in each cell subcluster, GSEA was performed by using the ranked expression values of DEGs, as implemented in the gseKEGG function of the clusterProfiler R package (v4.6.2). The cut-off value for the significant differences was determined using a threshold of *p* < 0.05.

### 3.11. Connectivity Map Analysis

Connectivity map methodology was employed to identify pharmaceutical agents with potential therapeutic repurposing capabilities in stroke. We uploaded 120 upregulated DEGs and the top 100 driver genes of microglia subcluster 2 to the CLUE database (https://clue.io, accessed on 13 July 2023) to query negatively connected drugs with the potential to reverse the transcriptional state of microglia subcluster 2. The 2D structures of the screened compounds were drawn by Chem3D software (v22.0.0.22).

### 3.12. Molecular Docking

To perform molecular docking, we obtained the protein structure files from the Protein Data Bank and used AutoDock Vina software (v1.1.2) to dock the ligands against the corresponding proteins. The docking results were then visualized using Pymol software (v2.5.5).

### 3.13. Statistical Analysis

All statistical analyses were conducted using R software (v4.2.3). In order to ascertain the time-dependent genes, the p-vector function, a component of the maSigPro R package (v1.70.0), was employed to calculate a regression fit for each gene across various experimental time groups. The identification of temporally DEGs was facilitated through the generalized linear model setting with *p* ≤ 0.05 after false discovery rate correction. The statistical significance of gene expression levels between normal subjects and stroke patients was determined using the Wilcoxon rank-sum test. The *p* values were adjusted for multiple testing by using the Bonferroni correction. DEGs for each cell cluster were discerned utilizing the FindAllMarkers function in the Seurat R package (v4.3.0). The Wilcoxon rank sum test with Bonferroni correction was applied for this statistical analysis. The significant GSEA gene sets were procured through the implementation of the permutation test, applying a false discovery rate correction to ensure statistical significance. The threshold for statistical significance was established at *p* < 0.05.

## 4. Conclusions

In conclusion, our study presents a nomogram model for stroke diagnosis and provides a potential molecule agent for stroke therapy. We employed WGCNA to identify gene modules associated with stroke and the maSigPro R package to seek the time-dependent genes in the progression of stroke. Three machine learning algorithms (LASSO, SVM-RFE, and Boruta) were further applied to identify the feature genes of stroke. A nomogram model based on these feature genes showed a higher net benefit after clinical intervention and enabled an accurate evaluation of patients with stroke. In scRNA-seq analysis, we found the crucial involvement of microglia in the pathologic progress of stroke. The trajectory and pseudo time analysis showed that microglia subcluster 0 would develop toward microglia subcluster 2 within 24 h from stroke onset. The GSEA analysis showed that the function of microglia subcluster 0 was opposite to that of microglia subcluster 2. The trajectory development and functional changes from microglia subcluster 0 to microglia subcluster 2 might cause subsequent stroke damage. AZ_628, which screened from CMap analysis, was found to have lower binding energy with Mmp12, Lgals3, Fam20c, Capg, Pkm2, Sdc4, and Itga5 in microglia subcluster 2 and maybe a therapeutic agent for the poor development of microglia subcluster 2 after stroke.

## Figures and Tables

**Table 1:** The information of the collected data.

Dataset ID	Control	Stroke	Tissue	Species	Data Type	Reference
GSE16561	24	39	Peripheral blood	human	Microarray	[54]
GSE58294	23	23	Peripheral blood	human	Microarray	[55]
GSE32529	6	8	Cerebral cortex	mouse	RNA-Seq	[56]
GSE112348	3	9	Cerebral cortex	mouse	RNA-Seq	[57]
GSE174574	3	3	brain	mouse	scRNA-Seq	[58]
PRJNA687414	6	6	microglia cells	mouse	RNA-Seq	[59]

## Data Availability

All relevant scRNA-seq, microarray data, and RNA-seq data for this study can be accessed in the GEO database (https://www.ncbi.nlm.nih.gov/geo/, accessed on 1 March 2023) or the EMBL-EBI database (https://www.ebi.ac.uk/, accessed on 1 March 2023) with accession number GSE174574, GSE16561, GSE58294 and PRJNA687414.

## Supplementary Materials

The following supporting information can be downloaded at: https://www.mdpi.com/article/10.3390/molecules28237704/s1. Table S1: Top 25 marker genes of Microglia subcluster 0 and 2; Table S2: Upregulated DEGs and driver genes of Microglia subcluster 2.
