# Supplementary material for: Integrative Analysis of Machine Learning and Molecule Docking Simulations for Ischemic Stroke Diagnosis and Therapy
Source: Molecules. 2023 Nov 22;28(23):7704. doi: 10.3390/molecules28237704 (PMC10707570; doi:10.3390/molecules28237704)
Supplement: Supplementary file 1 [file molecules-28-07704-s001.zip › molecules-2629761-supplementary.pdf]

Supplementary Material

1 Supplementary Tables

Table S1. Top 25 marker genes of Microglia subclusters 0 and 2

Marker genes of Microglia subcluster 0	Marker genes of Microglia subcluster 2
P2ry12, Selplg, Siglech, Gpr34, Crybb1, Pld4, Tmx4, Ltc4s, Tmem119, Serinc3, Vsir, Slc2a5, Hpgd, Itm2b, Cst3, P2ry13, Tgfbr1, Susd3, Marcks, Lpcat2, Rnaset2b, Rnase4, Cx3cr1, Csf1r, Ypel3	Spp1, Lgals3, Lpl, Lilrb4a, Lilr4b, Mmp12, Cd14, Cd72, Il1rn, Plin2, Ccl9, Adam8, Dab2, Id2, Sdc4, Fth1, Ccl4, Ctsb, Cd63, Capg, Anxa5, Ftl1, C3ar1, Cstb, Hmox1

Table S2. Upregulated DEGs and driver genes of Microglia subcluster 2

Upregulated DEGs of Microglia subcluster 2	Driver genes of Microglia subcluster 2
Abhd12, Adam8, Asph, Atf3, B930036N10Rik, Basp1, Bcl2a1b, Bcl2a1d, C1qa, C1qb, C3ar1, C5ar1, Calr, Capg, Ccl12, Ccl2, Ccl3, Ccl4, Ccl7, Ccl9, Cd14, Cd180, Cd52, Cd53, Cd63, Cd68, Cd72, Cd83, Cd84, Cd86, Cd9, Cndp2, Cotl1, Creg1, Csf1, Csf2ra, Cstb, Ctsa, Ctsb, Ctsc, Ctsd, Ctsl, Ctsz, Cxcl10, Cxcl16, Cyba, Cybb, Dab2, Edem1, Efhd2, Eif4a1, Emp3, Evi2a, Fam20c, Fcer1g, Fcgr1, Fcrls, Fth1, Ftl1, Fyb, Gadd45b, Gadd45g, Glipr1, Glrx, Gm10116, Gpr84, Grn, Gusb, Hmox1, Hspa5, Id2, Ifi204, Il1rn, Itga5, Itgb2, Lat2, Lgals3, Lgmn, Lilr4b, Lilrb4a, Lpl, M6pr, Manf, Milr1, Mmp12, Mnda, Mpeg1, Ms4a6d, Msr1, Npc2, Osm, Pdia3, Pdia6, Pkm, Plaur, Pld3, Plek, Plin2, Pmp22, Por, Prdx1, Rab7b, Rcan1, Rgs1, Rnf128, Rrbp1, Sdc4, Sdf2l1, Sgk1, Sirpa, Slc11a1, Slc15a3, Slc35f6, Slfn2, Spp1, Tgfbi, Tlr2, Tmem106a, Tnf, Tyrobp	Adam8, Adamts1, Adap2, Anxa5, App, Bcl2a1d, Brd2, C5ar1, Capg, Ccl4, Cd300a, Cd300lb, Cd36, Cdkn1a, Csf1, Ctsb, Ctss, Cx3cr1, Dab2, Dnajb9, Egr1, Emp1, Errfi1, Fam20c, Fli1, Foxn3, Gadd45b, Gapdh, Gas5, Gatm, Glrx, Gnl3, Gns, Gpr183, Havcr2, Hexb, Hist1h2bc, Hspa5, Icam1, Id2, Igf1, Il4ra, Iqgap1, Itga5, Itgam, Ldha, Lgals1, Lgals3, Lilr4b, Lilrb4a, Lpl, Mertk, Mfap3, Mmp12, Ms4a6d, Mt1, Mycbp2, Neat1, Nfkbia, Nfkbiz, Nlrp3, Nop56, Oxct1, Phlda1, Pkm, Plaur, Plk2, Por, Ppp1r15a, Ptp4a2, Rab20, Rab7b, Rbm47, Rin2, Rnf128, Sat1, Sdc4, Sdf2l1, Slc15a3, Slc37a2, Slc38a2, Slc6a6, Slfn2, Srgn, Tacc1, Taf1d, Tgfbi, Tgm2, Tmem106a, Tnf, Tob2, Trim30a, Tubb6, Ubash3b, Unc93b1, Vim, Wasf2, Wdr43, Zfand5, Zfas1
